# Applications of Artificial Intelligence in Dental Medicine: A Critical Review

**DOI:** 10.1016/j.identj.2024.11.009

**Published:** 2025-01-21

**Authors:** Symeon Sitaras, Ioannis A. Tsolakis, Marina Gelsini, Apostolos I. Tsolakis, Falk Schwendicke, Thomas Gerhard Wolf, Paula Perlea

**Affiliations:** a Private Practice, Thessaloniki, Greece; b Department of Orthodontics, School of Dentistry, Aristotle University of Thessaloniki, Thessaloniki, Greece; c Department of Orthodontics, C.W.R.U., Cleveland, Ohio, USA; d Department of Orthodontics, National and Kapodistrian University of Athens, School of Dentistry, Athens, Greece; e Department of Conservative Dentistry and Periodontology, Ludwig-Maximilians-University (LMU), Munich, North Dakota, Germany; f Department of Restorative, Preventive and Pediatric Dentistry, School of Dental Medicine, University of Bern, Bern, Switzerland; g Department of Periodontology and Operative Dentistry, University Medical Center of the Johannes Gutenberg-University Mainz, Mainz, Germany; h Department of Endodontics, Carol Davila University of Medicine and Pharmacy, Bucharest, Romania

**Keywords:** artificial intelligence (AI), decision-making, deep learning, dental medicine, dentistry, machine learning, review

## Abstract

**Introduction:**

Artificial intelligence (AI), including its subfields of machine learning and deep learning, is a branch of computer science and engineering focused on creating machines capable of tasks requiring human-like intelligence, such as visual perception, decision-making, and natural language processing. AI applications have become increasingly prevalent in dental medicine, generating high expectations as well as raising ethical and practical concerns.

**Methods:**

This critical review evaluates the current applications of AI in dentistry, identifying key perspectives, challenges, and limitations in ongoing AI research.

**Results:**

AI models have been applied across various dental specialties, supporting diagnosis, treatment planning, and decision-making, while also reducing the burden of repetitive tasks and optimizing clinical workflows. However, ethical complexities and methodological limitations, such as inconsistent data quality, bias risk, lack of transparency, and limited clinical validation, undermine the quality of AI studies and hinder the effective integration of AI into routine dental practice.

**Conclusions:**

To improve AI research, studies must adhere to standardized methodological and ethical guidelines, particularly in data collection, while ensuring transparency, privacy, and accountability. Developing a comprehensive framework for producing robust, reproducible AI research and clinically validated technologies will facilitate the seamless integration of AI into clinical practice, benefiting both clinicians and patients by improving dental care.

## Introduction

Over the past few years, there has been an outburst of research studies as well as applications of artificial intelligence (ΑΙ) technologies that have a significant impact on today's society and affect almost every aspect of human life. AI-based models are utilized in a wide range of applications from self-driving cars and voice assistants to medical diagnosis and financial analysis. In the field of medicine and specifically in dental science, AI algorithms have been implemented in almost every specialty, aiding the practitioner in the diagnosis, treatment planning, and decision-making process.

### Overview of AI

#### Artificial intelligence (AI)

**Fig. 1:**
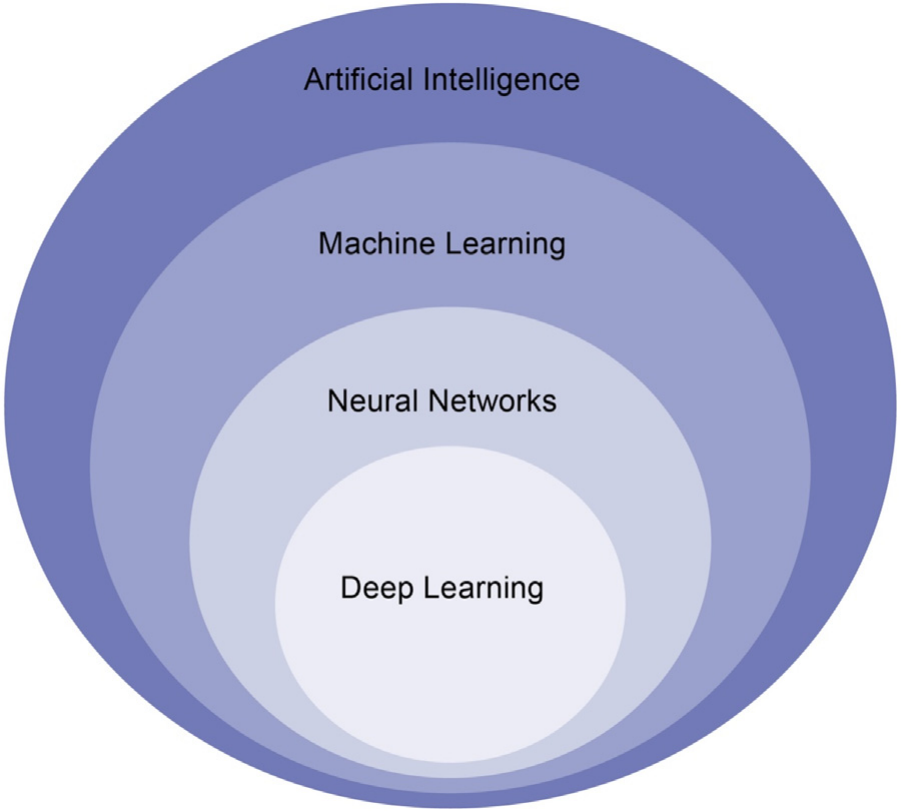
Artificial intelligence and its subfields.

AI is a field of computer science and engineering that focuses on creating machines capable of performing tasks that typically require human intelligence, such as visual perception, speech recognition, decision-making, and natural language processing. The history of AI can be traced back to the mid-20^th^ century when a group of researchers coined the term "artificial intelligence" and began to explore the possibility of creating intelligent machines. AI became increasingly popular, particularly in the fields of medicine and healthcare, during the early 21^st^ century as machine learning was able to solve numerous academic and industrial problems thanks to the utilization of advanced computer hardware, novel methodologies, and the accumulation of vast amounts of data.^1^ Artificial Intelligence is a broader term that incorporates the subfields of Machine Learning (ML), Neural Networks (NN), and Deep Learning (DL) (Figure 1).

#### Machine learning (ML)

ML is the development of computer systems that use algorithms and statistical models to find structures and patterns within data. These algorithms can learn and adapt each time new data are introduced and consequently improve over time, without human input. ML techniques include linear regression, logistic regression, naive Bayes, decision tree, nearest neighbor, random forest, discriminant analysis, support vector machine, and neural network (NN).^2^

#### Neural network

**Fig. 2:**
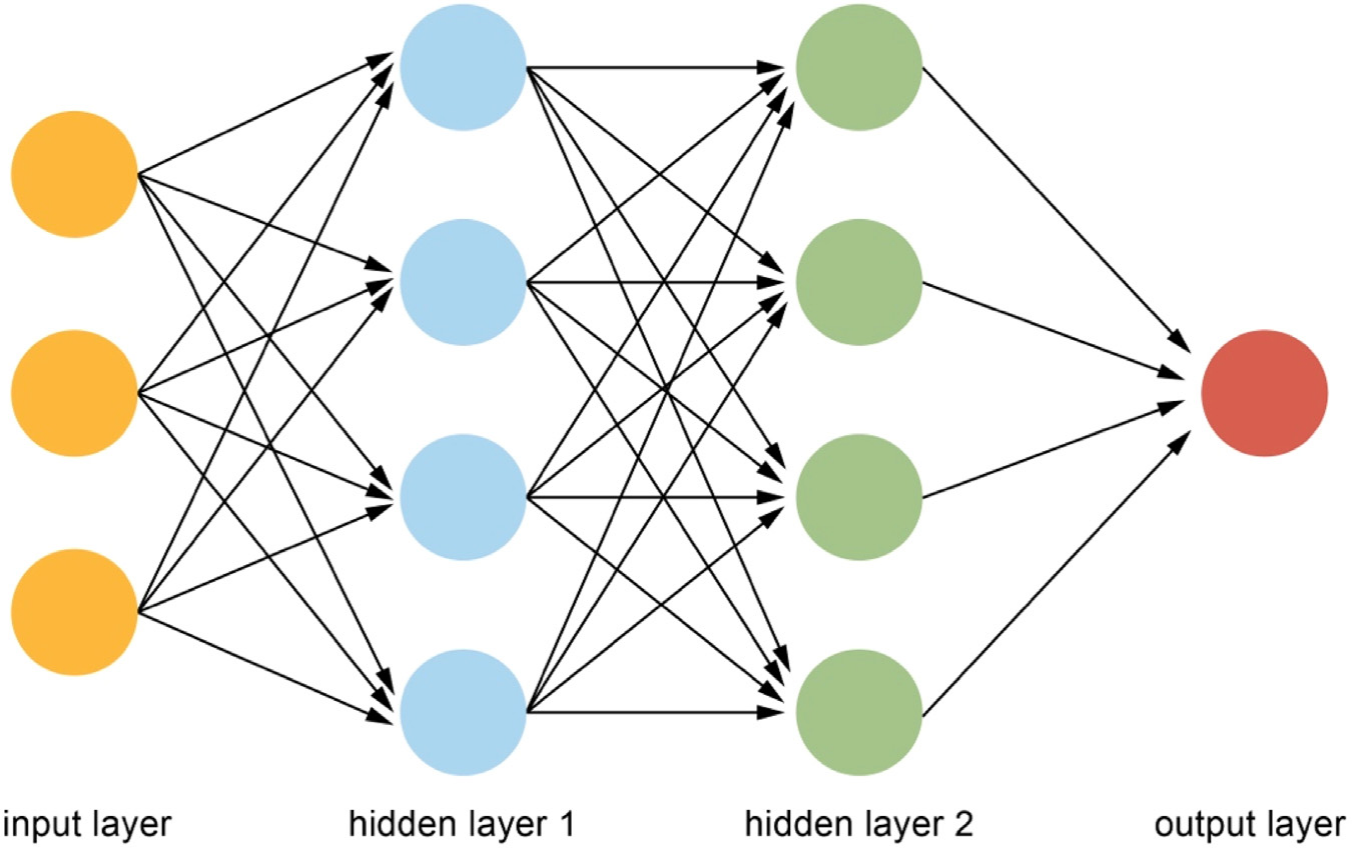
Neural network structure.

A neural network (NN) is a construct of algorithms that compute signals via artificial neurons through a process that mimics the human brain and the biological NN. Through interconnection, NN can explore nonlinear information in the data, and recognize underlying patterns in input information and respond with an appropriate output. A typical NN has an input layer, consisting of one or more input variables, one or more hidden layers or nodes, and an output layer, which has one or more neurons. The association between the input variables and the outcome is depicted through the hidden layers (Figure 2).^3^

#### Deep learning (DL)

DL is a subfield of ML, based on artificial neural network (ANN). The most common DL architecture is the convolutional neural network (CNN). It uses multiple processive layers to progressively extract higher-level features from the raw input and detect nonlinear patterns in the data, a process common to any artificial neural network (ANN). What distinguishes convolutional neural networks (CNNs) is their use of the convolution operation, which enhances feature extraction. CNNs are mainly used for the analysis of complex imagery data. By using multiple hidden layers, they can extract and detect image features like edges, corners, and macroscopic patterns.^4^^,^^5^

### Data analytics and clinical decision support systems (CDSSs) in healthcare

**Fig. 3:**
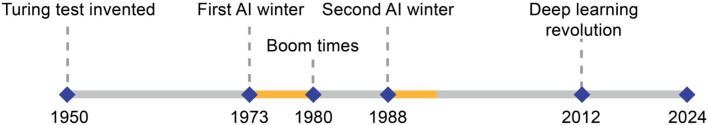
Timeline of artificial intelligence.

We are currently experiencing a digital revolution that has largely automated and reshaped the medical as well as the dental field. The vast amount of data collection and processing and the advanced computing solutions that have risen over the last 20 years boosted the research and gave birth to novel applications that utilize ΑΙ algorithms creating new pathways for modern healthcare delivery (Figure 3). The progress of information technology (IT) promoted the explosion of stored data and the adoption of electronic health records, paving the way toward a more digitalized approach. The stages that characterize digital health care are data collection, data sharing and data analytics.^6^ Advancements in AI technologies have enabled the next stage of digitization to emerge. By exploring associations and effectively analyzing patterns within the data, big data analytics have the potential to enhance care provision and lower costs^7^. Additionally, the fusion of IT with the healthcare industry gave birth to clinical decision support systems (CDSSs).^8^ CDSSs are computer programs that use high-end computational resources and analytical methods to provide expert support to medical professionals and improve the clinical workflow. CDDSs have been a part of medicine for the past several decades, improving clinical decision-making,^9^ and supporting the delivery of quality care,^10^ whilst reducing the frequency and consequences of errors. An integral part of AI applications in healthcare involves the use and development of CDSSs.^11^

### Development of AI models

The development of an efficient AI model requires the collection of high-quality data sets. Raw data should be cleaned and processed, with data mining focused on extracted valuable insights rather than simply cleaning. This process helps to remove redundant features and duplicates, manage missing data, and cross-relate data from different sources.^12^ After the collection and refinement of the data, an effective methodology must be selected. ML strategies involve supervised and unsupervised learning.^13^^,^^14^ The most common learning strategy is supervised learning. In supervised learning, predetermined input and output data sets are provided to train the algorithm (training set). Then, after the adjustment of the parameters (validation set), the model is tested (test set). The aim is to design a model that when given new input data, can predict accurately the outcome. Supervised learning is more commonly used for image analysis in healthcare due to its ability to provide clinically relevant results. However, in areas such as text, speech, and certain healthcare applications, unsupervised learning is becoming increasingly prevalent. In unsupervised learning, algorithms work with unlabeled data to identify hidden patterns and relationships between features, operating without predefined outcomes.

### AI and dental applications

AI innovative new techniques and applications have also taken by storm the dental community.^15^^,^^16^ Research studies around new applications have exploded, especially in the last decade, covering various domains of the field. Advanced diagnostic and treatment tools that utilize AI algorithms have been developed to support clinicians in providing optimum and personalized dental care. These models exhibit promising results, sometimes outperforming experienced specialists, thus creating new possibilities for a more structured and time-efficient approach towards dental science. This rapid advancement has raised not only high expectations but also certain concerns regarding the planning of the research and the applicability of some applications. ΑΙ historically has lived through phases of generalized hype and excitement, as well as phases of disappointment, often referred to as AI “winters”. During those phases both the interest and the funding were significantly decreased, resulting in the overall reduction of AI developments. As we are now experiencing a rapid increase in AI research it is also crucial to address the limitations that several studies present along with the ethical dimensions of the subject, to increase the reliability and robustness of AI-related studies and ultimately avoid another “winter”. The aim of this study is to a. review the existing applications of artificial intelligence in dental medicine taking into consideration all the main specialties and b. examine the current perspectives and challenges of these applications, considering the limitations, ethical complexities, and important methodological guidelines for AI-related studies.

## Applications of AI in dentistry

### AI and orthodontics

In orthodontics and dentofacial orthopedics, AI algorithms have been utilized in various applications over the last 2 decades paving the way for an automated digital diagnostic and prognostic approach in clinical care. Cephalometric analysis is an integral part of orthodontic diagnosis and treatment planning, playing a pivotal role in the success of the treatment. The assessment of lateral cephalograms is mainly performed for sagittal evaluation of skeletal and dentoalveolar relationships, analysis of the soft tissues of the face, changes that occur during treatment procedures, and estimation of growth patterns and development. The conventional way of cephalometric analysis is through manual landmark identification, which is time-consuming and associated with a high potential for error and bias. Due to the digital evolution of the last decades and the progress of computational techniques, we are now pacing rapidly towards a fully automated analysis of the cephalograms. Recently developed AI-based models, utilizing ML and DL algorithms, have demonstrated consistently high accuracy in detecting cephalometric landmarks in 2D images. However, their performance in 3D imaging remains an area requiring further improvement. These tools hold promise for assisting practitioners by minimizing subjective errors and saving time.^17^, ^18^, ^19^, ^20^, ^21^, ^22^ Despite the high expectations for the daily use of DL algorithms in clinical practice, the overall evidence is of limited generalizability and robustness.^23^

The patient's stage of skeletal growth is of critical importance in orthodontics because it determines the optimal time for intervention and in consequence the achievement of a successful morphologic result within optimal treatment duration. A valid diagnostic method used widely for the identification of the growth curve and the skeletal maturity of the patient is the radiographic evaluation of the cervical vertebrae maturation (CVM). However, it requires not only specialized skills but also a great deal of time. Addressing a need for an automated and accurate analysis of the CVM stage, AI algorithms have been tested on lateral cephalograms demonstrating satisfying performance.^24^, ^25^, ^26^, ^27^, ^28^, ^29^, ^30^, ^31^

Besides landmark tracing and CVM assessment, DL CNNs have also been used on lateral cephalometric radiographs for the automated diagnosis of adenoid hypertrophy in children, exhibiting accurate results and reliable performance.^32^ Artificial intelligence is also being used in orthodontics for teeth segmentation, superimposition of dental arches, and airway analysis on 3D imaging. A very important usage of artificial intelligence software is the automatic tooth segmentation in digital dental models. DL-based automatic tooth segmentation of a digital model demonstrates a great success rate, accuracy, and efficiency in tooth segmentation. Therefore, it may be used for orthodontic diagnosis and appliance fabrication. A great application of artificial intelligence in orthodontics is the superimposition of dental casts to assess tooth movement during or after orthodontic treatment. According to the present literature, in comparison to other software types, semiautomatic best-fit registration software regularly shows outstanding agreement in superimpositions. Compared to other methods, automatic best-fit registration software consistently showed superior agreement for mandibular superimpositions. The quantification algorithm used in superimposition investigations can be credited with the accuracy of digital model superimpositions for tooth movements. Lastly, artificial intelligence can be used to automatically segment and analyze the airway. Due to a successful AI technique, the pharyngeal airway may now be automatically segregated from CBCT images. According to the existing literature, automatic segmentation techniques can be effectively employed in clinical settings to assist with tasks like orthodontic diagnosis and appliance fabrication, offering high accuracy and efficiency compared to traditional methods; this is because it seems to be quick and simple to use while also measuring the airway with great accuracy.^33^, ^34^, ^35^ Another important aspect regarding the orthodontic treatment plan is whether to extract permanent teeth or not. The decision to extract requires the analysis of multiple variables including cephalometric measurements, clinical findings (maxillary and mandibular crowding, overjet, overbite), periodontal condition, facial esthetics (lip protrusion) as well as the patient's systemic health.^36^^,^^37^ Be that as it may, this decision depends entirely on the practitioner's training, clinical experience, and treatment philosophy and that is why there is a considerable disagreement regarding the judgments on tooth extractions delivered by orthodontists. To bridge that gap, AI-based models, using ML algorithms have been utilized on clinical, radiographic, and demographic data to facilitate the clinical decision-making process. The models demonstrated high accuracy and efficient performance not only for the binary decision extraction/no extraction but also for other possible outcomes.^38^, ^39^, ^40^, ^41^, ^42^, ^43^

### AI and periodontology

Periodontitis is a bacterial-driven chronic inflammatory disease of the tissues surrounding and supporting the dental root. The continuous progression of periodontitis results in the destruction of all periodontal tissues including the alveolar bone, gingiva, and periodontal ligament around the tooth. Early detection and correct identification of periodontal diseases could avert the onset of tooth loss, prevent systemic diseases related to periodontitis, and reestablish patients’ oral health. To develop efficient diagnostic models for the classification of periodontitis, researchers utilized ML algorithms. The algorithms were trained and tested on clinical indexes, radiographic measurements, demographic data as well as immunologic parameters. The models demonstrated satisfying performance in diagnosing the grades of periodontal disease,^44^^,^^45^ classifying patients belonging to either aggressive periodontitis or chronic periodontitis,^46^ assessing the progression of the disease, and determining its severity degree.^47^ AI research in periodontology also includes the development of DL models, using convolutional neural network (CNN) algorithms. Deep CNNs were applied on panoramic radiographs for the detection of periodontal bone loss^48^, and additionally on periapical radiographs for the diagnosis of periodontally compromised teeth, achieving high discriminating and diagnostic ability, matching that of trained specialists.^49^ Periodontal disease is linked to systemic health diseases, such as cardiovascular disease, stroke, osteoporosis, and diabetes. In a novel approach, a DL convolutional neural network was combined with intraoral fluorescent biomarker imaging and clinical examinations, developing an automated process for oral health screenings and the correlation of systemic health conditions and periodontitis.^50^

### AI and restorative dentistry and prosthodontics

Treatment planning is one of the most challenging aspects of clinical practice, requiring an accurate diagnosis and evaluation of the prognosis. The prognosis of the teeth depends on several patient-specific variables as well as a multidisciplinary analysis of the oral structure. An AI-based system, using ML algorithms, was designed to facilitate clinical decision-making regarding tooth prognosis, taking the ideal treatment plan into account.^51^ Following the correct assessment of prognosis, the subsequent decision for teeth extractions is also a critical part of the developing treatment plan and its long-term success. In another approach, a clinical decision support (CDS) system utilized ML algorithms on electronic dental records, to determine appropriate tooth extraction therapy in clinical situations. The model achieved high performance, outperforming 2 trained prosthodontists.^52^ Restorative materials, such as composite resins (CR), amalgams, metals, and ceramics are routinely used in clinical practice. However, the lifespan of these restorations is limited, depending on the material used and the characteristics of the tooth's cavity and remaining walls. In that scope, a case-based reasoning tool, utilizing a neural network for the classification of CR and amalgam restorations, was developed to predict the longevity of these restorations.^53^ As CAD/CAM restorations are used more and more in clinical practice, the need to prevent debonding and improve the survival rate of these restorations is increasing. Addressing this issue, a DL CNN was used to predict the debonding probability of CAD/CAM CR crowns, demonstrating considerably accurate performance.^54^ In restorative dentistry, matching the color of a ceramic restoration with a natural tooth is a challenging task. To this end, a method that enhances the prediction and precision of color matching, using a genetic algorithm and back propagation neural network, was developed.^55^ The aesthetic outcome of the maxillary anterior region is of critical importance for both the patient and the dentist. To achieve a better outcome, it is essential that the dentist performs a comprehensive examination and analysis of the aesthetic zone. An aesthetic region teeth segmentation algorithm based on curvature analysis and active contour was designed, to facilitate automated smile analysis, exhibiting high accuracy rates. Due to the limitations of the model, further research is needed.^56^

### AI and oral and maxillofacial surgery

Oral squamous cell carcinoma (OSCC) constitutes the major neoplasm of the head and neck region, exhibiting a quite aggressive nature, often leading to unfavorable prognosis. Although current advances in treatment protocols have successfully tackled the disease, a substantial percentage of affected patients suffer from relapses, due to the deeply infiltrated nature of these tumors.^57^ Early identification of a potential disease reoccurrence and accurate modeling of the disease progression can be very beneficial for the prognosis of the patient.^58^

To this end, ML algorithms have been applied to clinicopathologic data, imaging data, and genomic markers for the development of an oral cancer prognostic model, demonstrating superior performance with measurable improvements in accuracy, sensitivity, and specificity compared to current standard methods.^59^^,^^60^ In addition, most cases of OSCC are in an advanced stage when diagnosed, which significantly affects the survival rate after the surgical treatment. Early detection of OSCCs could lead to an overall better curative outcome as well as lower recurrence rates. In a novel approach, DL CNNs exhibited satisfying performance when applied on laser endomicroscopic images for the automatic classification of cancerous lesions.^61^ DL CNNs have also been applied to CT scans for the evaluation of extra-nodal extension of cervical lymph node metastases in patients with OSCC.^62^

Bisphosphonates are routinely prescribed for the management of osteoporosis, reducing fracture risk at various skeletal sites. However, some unexpected possible adverse effects have been reported, including osteonecrosis of the jaw. Tooth extraction is considered one of the risk factors for bisphosphonate-related osteonecrosis of the jaw (BRONJ) and its avoidance is recommended.^63^, ^64^, ^65^ Nevertheless, in some cases is necessary due to the possibility of more severe infections. Five types of ML models were designed to predict the occurrence of BRONJ associated with dental extractions, demonstrating considerable accuracy.^66^

In implant dentistry, the assessment of bone density is an integral part of the surgical treatment plan, influencing the overall success rate of the treatment.^67^ A new approach introduced a CNN-based method for the automatic classification of the alveolar bone density utilizing 3D volumetric data in CBCT images.^68^ Extraction of impacted mandibular third molars is one of the most common oral surgical procedures. The amount of postoperative facial swelling that follows third molars removal varies depending on gender, age, the degree of impaction, surgical technique, and operating time.^69^^,^^70^ To predict postoperative facial swelling, an artificial neural network was trained and tested on patients’ clinical and demographic data, achieving a highly accurate performance.^70^

### AI and endodontics

Vertical root fractures (VRFs) are one of the most challenging situations in clinical practice not only to diagnose accurately but also to treat conservatively. Besides clinical signs and symptoms, the diagnosis of a VRF is based on the radiographic identification of a fracture line. Although detectability of VRFs is reported to be higher in CBCT images rather than conventional periapical or panoramic radiographs,^71^^,^^72^ it remains a difficult task and depends on the diagnostic performance of these radiographs as well as the experience of the dentist.^72^ Not to mention that the increased radiation exposure, the high cost and artifacts resulting from root canal treatment materials^73^ constitute an additional barrier to the everyday use of CBCT scans. Taking all the above into consideration, in the last decade, researchers developed ML models that can automatically detect and diagnose VRFs on panoramic, periapical, and CBCT radiographic images.^74^, ^75^, ^76^

Accurate working length determination is a crucial factor for the success rate of a root canal treatment.^77^ The correct working length, where the biomechanical preparation and the root canal filling should terminate, is at the minor apical foramen.^78^ In a new approach for locating the minor apical foramen, an artificial neural network was trained and tested in radiographs of single-rooted teeth both *ex vivo* and in human cadavers.^79^^,^^80^ The model demonstrated a highly accurate performance, significantly outperforming endodontists’ estimations.

The distal root of the mandibular first molar occasionally has a second root, which if overlooked can affect the outcome of the endodontic therapy. A DL system was used on panoramic radiographs to assess the root morphology and determine the number of distal roots of mandibular first molars, achieving high diagnostic performance.^81^

Apical periodontitis is defined as an inflammatory process around the apex of the tooth root and is detected radiographically as apical lesions (a widened periodontal ligament or a clearly detectable lesion). A deep convolutional neural network was applied on panoramic radiographs to detect and classify apical lesions, demonstrating satisfactory discriminatory ability.^82^

One of the most crucial parts before an endodontic therapy is the correct assessment of the case difficulty and its subsequent prognosis. The American Association of Endodontists (AAE) case difficulty assessment form is a standard form that provides a template for general dentists to objectively assess the difficulty of a case and decide for a referral to a specialist or not. ML algorithms were trained and tested on a dataset of endodontic cases using the AAE form to automatically estimate the difficulty level of these cases, exhibiting an accurate performance. The model can be employed in clinical practice, increasing the speed of decision-making and referrals if necessary.^83^

### AI and oral and maxillofacial radiology

DL-convolutional neural networks (CNNs) have been applied in numerous applications in oral and maxillofacial radiology, as they represent a state-of-the-art approach for recognizing and analyzing patterns in various radiographic images. Dental caries is one of the most prevalent oral health problems. Conventional caries detection involves oral examination and the use of the dental probe for clearly visible lesions, and dental radiographs for detecting hidden or inaccessible lesions. However, early detection of caries, which would reduce the need for invasive procedures, could benefit from the introduction of new methodologies and tools.^84^ In that scope, in the last years several researchers have aimed to develop efficient models, using DL algorithms, mainly CNNs, for caries detection. Periapical radiographs,^85^ bitewings,^86^ and near-infrared transillumination images^87^^,^^88^ have been used for training and testing these algorithms. The AI-based models demonstrated satisfying performance, suggesting that these applications may find use in clinical practice assisting dental practitioners and increasing the accuracy of caries detection.

Another field in oral radiology where ML algorithms have been utilized is for the detection of osteoporosis. Dental panoramic radiography is widely considered as a cost-efficient way for detecting osteoporotic changes. Panoramic mandibular indices such as gonion index, mandibular cortical index, mandibular cortical width, etc. have been developed to assess the quality of mandibular bone mass and detect signs of resorption. Early detection of osteoporosis, especially to asymptomatic patients, is a difficult task for the general dentist. To that end, deep CNN methods have been designed to process efficiently panoramic X-ray images and provide information to clinicians for early identification of osteoporosis.^89^, ^90^, ^91^. Thus, contributing to the early referral of the patient to appropriate medical professionals.

Conventional 2-dimensional panoramic radiographs are the most routinely used imaging technique to assess the orientation of mandibular third molars and their relationship to the mandibular canal. In a promising approach, deep-learning CNNs were applied to orthopantomograms for an automated calculation of the proximity of mandibular third molars to the inferior alveolar nerve (IAN).^92^ Consequently, an AI-based method was created, to assess the risk of IAN injury that follows third molar removal.

In another innovative research CNNs were used to automatically measure the angulation of mandibular third molars to predict their eruption chances.^93^

Temporomandibular joint osteoarthritis (TMJOA) is an important subtype of temporomandibular disorders, and its pathology includes progressive cartilage degradation, masticatory dysfunction, and pain.^94^ TMJOA is confirmed by structural bony changes observed on computed tomography (CT) scans, cone beam CT (CBCT) images, and panoramic radiographs. Taking into consideration that accurate diagnosis of TMJOA during the early stages of the disease is both challenging and crucial, DL algorithms have been developed to automatically detect and classify TMJOA in CBCT images^95^^,^^96^ and orthopantomograms (OPGs),^97^ supporting clinicians in the decision-making process.^98^

ML and DL algorithms have also been successfully implemented for diagnosing maxillary sinusitis on panoramic radiographs^99^^,^^100^ and for identifying Sjogren's syndrome on CT scans,^101^ demonstrating high diagnostic performance as well as comparable efficiency with that of radiologists. They have yielded a highly accurate performance for detecting dental restorations^102^ and recognizing supernumerary teeth^103^ on panoramic radiographs. Lastly, CNN-based systems have achieved an excellent performance for teeth detection and segmentation on panoramic radiographs to automate dental charting purposes and improve the clinical workflow.^104^, ^105^, ^106^, ^107^

### AI and pediatric dentistry

Detection and control of dental plaque is a critical aspect of preventing oral diseases and maintaining children's oral health. Addressing the need for a cost-effective and convenient technique to objectively detect and quantify dental plaque, an innovative AI model was designed to detect plaque on primary teeth. Deep-learning CNNs were trained and tested on a dataset of intraoral photos of deciduous teeth. The model showed clinically acceptable performance, like that of an experienced pediatric dentist.^108^ Panoramic radiography is used routinely in pediatric patients, mainly for evaluation of the stages of dentition and dental abnormalities. Taking a step towards a digital diagnostic approach, a DL algorithm was used on panoramic radiographs for the automated detection and numbering of deciduous teeth, exhibiting a highly accurate performance.^109^

### AI and forensic odontology

Forensic odontology is a specialized field of dentistry that involves the management, examination, evaluation, and presentation of dental evidence in criminal or civil proceedings, all in the interest of justice. It also plays a pivotal role in the identification of the victims of multifatality disasters (natural disasters, nuclear disasters, etc.) and generally in cases of decomposed, charred, or skeletonized bodies.^110^ In that frame, age estimation, gender determination, and facial reconstruction are subdisciplines of the forensic sciences that constitute an important part of the identification process, especially when information relating to the deceased is unavailable. In recent years some researchers focused on developing automated identification models using artificial intelligence algorithms for the scope of enhancing these forensic processes.^111^^,^^112^ The models exhibited promising performance, opening a new field for AI research.

## Current perspectives and challenges

AI-related studies and research in the field of dentistry are increasing rapidly in the last decade.^113^ The adoption of ML algorithms has stimulated the development of automated diagnostic and prognostic models that support clinicians in the provision of personalized, high-quality dental care and simultaneously relieve the workforce from laborious tasks.^114^ AI-based applications signal the digital transformation of dental medicine and can contribute to the management of current and upcoming challenges in oral healthcare. Nevertheless, despite the high expectations and all the potential around AI research, there are also concerns regarding the applicability and generalizability of the results that several studies present as well as the ethical challenges that coexist with them.^114^ Until recently, the widespread implementation of AI in routine dental practices was not technically possible or financially viable, so the potential of AI has not been fully realized in the field.^115^ The first implementations of CNNs are just entering the clinical workspace, but on a large scale, AI applications have not been integrated into routine care.^116^ Many studies suffer from methodological weaknesses and reporting limitations and as the spectrum of applications in dentistry broadens, it is crucial to examine these flaws and explore a more robust way of conducting AI research.^117^

### Addressing the limitations

#### Data collection and spectrum bias

The data collection methodology is of critical importance since the various data resources constitute the elements on which the AI algorithms are trained, validated, and tested. Authors and reviewers should be aware of certain pitfalls and biases that occur in AI research.^118^^,^^119^ An imbalanced data collection process or insufficient reporting of the data resources may result in a dataset that does not entirely represent the possible clinical and demographic characteristics of the task at hand. To alleviate spectrum bias, data should be of sufficient quality and representative of the target population and settings of the application.^118^

#### Overfitting and selection or discriminatory bias

Using narrow and limited datasets, which is common in dental research, can lead to the development of algorithms that appear efficient and accurate but fail to generalize well to new, unseen data. To ensure more robust models,^120^ researchers are advised to use larger and more diverse sample sizes. Additionally, data snooping bias can occur when similar or identical data is used in both the training and test sets, resulting in artificially inflated algorithm performance.^114^, ^119^ In such cases, the model may simply memorize the data from the training set, leading to misleadingly high performance on the test set. To avoid this, it is recommended that AI algorithms be validated using an independent, external dataset. Additionally, when splitting data for training and testing, care should be taken to account for clustering effects, such as ensuring that multiple images from the same patient are not split across both sets, which could artificially inflate performance metrics.^120^ Inaccessible, missing, or inadequate data, from electronic health records, could impair the sampling process. By interpreting only the available data, algorithms may exclude individuals with missing data and typically overrepresent the majority while underrepresenting minorities. It is of critical importance that AI studies ensure diverse and representative datasets to avoid the risk of bias and be reliable across different populations.^120^

#### “AI-chasm” and lack of transparency

Describes the gap between an AI-based model and its practical real-world application. The metrics chosen to optimize the model along with an unclear training and validation strategy may not reflect clinical applicability.^119^ In other words, designing an accurate system that does not necessarily mean that is clinically applicable. Designing an effective algorithm on a small dataset from a specific population differs significantly from developing an algorithm that can be implemented across different populations and clinical settings.^121^ The difficulty in interpreting and explaining how highly complex algorithms make certain decisions has led many researchers to acknowledge that neural networks mostly remain a “black box”.^122^ Failure to explain understandably the decision-making process that ML models follow impedes practitioners’ trust in clinical AI. Given that many AI applications use highly complex prediction models, researchers should aim to provide elements of explainable AI in their studies,^123^ so that patients and healthcare providers can understand how they work and how they make decisions. As a result, there has been a growing interest in creating techniques to display, clarify, and comprehend DL models.^124^

### Planning and reporting AI research

Inconsistent data quality, risk of bias and limited evidence to support the clinical effectiveness of AI are significant issues that impair the quality of the studies and compromise the effective integration of the applications into routine clinical practice.^125^ The impressive array of studies necessitates that a more robust, high-quality methodological process for planning, conducting, and reporting of AI research should be followed.^117^^,^^123^ Despite the limited amount of published randomized clinical trials in AI, authors are encouraged to consult guidelines like the extensions of the CONSORT (**CON**solidated **S**tandards **O**f **R**eporting **T**rials)^126^ and SPIRIT (**S**tandard **P**rotocol **I**tems: **R**ecommendations for **I**nterventional **T**rials)^127^ statements in AI for reporting RCTs and RCTs protocols respectively. Additional guidelines, although they don't focus on AI, that could be of use include the TRIPOD (**T**ransparent **R**eporting of a multivariable prediction model for **I**ndividual **P**rognosis **O**r **D**iagnosis)^128^ statement that is designed to improve the reporting of studies developing a prediction model, the STARD (**STA**ndards for **R**eporting of **D**iagnostic accuracy studies)^129^ statement that aims to enhance the quality of reporting of diagnostic accuracy studies. Researchers are also advised to employ tools like the QUADAS-2 (**QU**ality **A**ssessment of **D**iagnostic **A**ccuracy **S**tudies)^130^ that is applied to systematic reviews or the PROBAST (**P**rediction model **R**isk **O**f **B**ias **AS**sessment **T**ool)^13^1 to estimate and potentially mitigate the risk of bias.

A recently published checklist that is addressed to authors, reviewers, and readers of dental research in AI can serve as an instructional map to assist researchers design more robust and transparent studies, raising the standards in the field.^132^

Indicatively, it is worth noting that when considering the development of an AI-based model, researchers are advised to:

• define the meaning and scope of the application (diagnostic or prognostic),
• use high-quality, adequately sized datasets that are as heterogeneous as possible to strengthen generalizability,
• construct a solid reference test, probably using several independent annotators to label the data,
• consider clustering of teeth or patients to minimize data snooping bias,
• use an independent external dataset to test the algorithm to ensure generalizability,
• assess the computational resources needed, as they play a pivotal role in the processing of the data,
• compare the model against relevant alternatives (dental experts or other models).

As far as the reporting methodology, it is recommended to:

• provide an overview of the study goal,
• describe the structure of the model (input-output layers, etc.)
• report all the data resources and explore the possibility of bias,
• describe the chosen method to train, validate, and test the model,
• describe the results and clarify the performance metrics on all data partitions,
• explain the clinical applicability of the model,
• discuss not only the strengths but also the limitations of the application [132].

Planning reproducible and transparent studies and designing effective and applicable models are of utmost importance to foster trust in clinical AI and utilize the benefits of its use. To this end, it is also critical to identify the ethical challenges that emerge in AI research.^133^

### Ethical concerns

A major concern that is frequently addressed, especially in the era of digitalization with the adoption of vast amounts of electronic health records, is the patient's privacy and confidentiality.^134^^,^^135^ As AI systems are integrated into healthcare systems, there is an increased risk of data breaches or misuse of patient information. This could lead to unintended harm to patients and erosion of trust in healthcare providers. Furthermore, there are concerns about accountability and transparency in the decision-making process.^119^^,^^122^ If an AI algorithm makes a mistake or produces an unexpected outcome, it may be difficult to determine who is responsible and how to rectify the situation. This lack of accountability could undermine patient trust in the healthcare system. Other concerns that need to be tackled include the integration of AI in clinical practice, as a supporting tool and not a substitute for the clinician, the role of AI in the education and training of medical and dental students^136^ as well as the legal conflicts that may arise with the use of AI in healthcare.^134^ An additional concern, in terms of geographic distribution and AI-related studies, is that data show a prominent representation of more economically developed countries and an underrepresentation of certain geographic areas, indicating that AI advances are not accessible to all.^137^ By sharing more openly their data and algorithmic codes, research teams can contribute to moderating this phenomenon. When considering ethics and AI research in dentistry, 6 main principles arise prudence, privacy, responsibility, democratic participation, solidarity, and equity.^138^ It is important to highlight that most of the studies do not provide access to the data nor to the code developed, limiting the overall reproducibility of corresponding research in the field. Most of the studies are validated only internally, thus increasing the bias associated with them. And lastly, only a small number of studies report ethical concerns, depicting that the medical community remains widely uninformed about the ethical complexities that emerge around AI research.^138^

## Discussion

### Benefits of AI

Artificial intelligence (AI) is already shaping the dental landscape by revolutionizing the way professionals diagnose and treat oral health problems. Some of the primary ways in which AI is making an impact in dentistry are:

#### Accurate diagnosis and personalized treatment planning

AI algorithms can analyze dental radiographic images, such as panoramic and cephalometric radiographs, CBCT scans, periapical radiographs, and bitewings, to detect oral health problems like caries, periodontal disease, root fractures, TMJOA, oral cancer, etc. These powerful tools assist clinicians in making more accurate diagnoses and providing more effective treatments. By analyzing patient data, such as medical history, clinical indexes, and radiographic features, AI algorithms can consider the patient's unique characteristics and recommend the best course of treatment. This can help practitioners provide more targeted and personalized treatments.

#### Prognostic analysis and Improved efficiency

By identifying patterns in patient data AI algorithms can also inform treatment decisions, prevent oral health problems, or predict treatment outcomes. Highly accurate prognostic models have been developed to estimate tooth decay, periodontally compromised teeth, the need for orthodontic extractions, the risk for postoperative facial swelling following third molar removal, the risk for BRONJ, etc. These models can assist professionals in the treatment planning and decision-making process. AI can help dental practices operate more efficiently by automating tasks like appointment scheduling, and patient follow-up, while also improving the accuracy of dental charting and treatment recommendations. AI can help to reduce the burden of administrative tasks, freeing up time for dentists to focus on providing high-quality patient care.^139^

### Fostering trust in AI

As we discussed, besides the benefits that the advancement of AI offers in the field of dentistry, a few concerns have also emerged along with them. Insufficient data collection processes, risk of bias, reporting limitations, lack of transparency, and lack of understanding of how to effectively incorporate AI into the clinical arena, are significant issues that need to be addressed. To overcome these barriers and produce more robust and reproducible results a multifaceted approach is required. It is strongly recommended that studies in AI follow methodological and ethical guidelines that include standards for data quality, transparency, privacy, accountability, and replicability.^123^^,^^132^ Additionally, it is essential to establish a comprehensive framework for data sharing and collaboration, invest in the development of AI technologies that are tailored to healthcare needs, and promote a culture of innovation and experimentation in healthcare organizations.^140^ Discussing the concerns that arise, exploring possible solutions, and developing clinically validated AI technology, will ultimately facilitate the integration of ML models in the clinical workflow and help both practitioners and patients understand the role AI can play in healthcare.

### Reshaping dental health landscape

The rising costs of dental care and a growing number of patients unable to access necessary treatments, coupled with the increasing healthcare needs of an aging population, demonstrate the pressing need for a new and sustainable model of dental care.^141^ The integration of digital technology presents a highly encouraging tactic for transforming the field of oral healthcare. Utilizing modern technology, along with the advancement of ML algorithms in dental medicine, presents an occasion to shift away from a "disease-focused" model and towards a patient-centered approach to care, which represents a significant change. To enhance patient-centered outcomes, dental research should prioritize connecting oral and general health and advancing personalized medicine. Instead of solely generating scientific publications, dental research should focus on delivering a tangible impact to society by implementing changes to clinical protocols. ^142^ The appropriate integration of AI into clinical workflow can provide clinical patterns and insights beyond human capabilities and reduce the burden of integrating vast amounts of health data into clinical practice. This can free clinicians to focus on placing insights into clinical context and attending to patient's needs to achieve optimal health.^125^^,^^139^ A new popular term has emerged: augmented intelligence.^125^^,^^142^ This refers to the integration of digital tools with human qualities and capabilities to enhance dental and oral healthcare and ultimately improve quality of life. The blending of AI and human intelligence, or augmented intelligence, is considered the most effective method for achieving the core objective of healthcare.

## Conclusions

A variety of diagnostic and prognostic AI-based models have been developed in almost every specialty of dental medicine. These models utilize ML algorithms and have the potential to revolutionize the way we perform treatment planning as well as enhance the way we provide nonbiased, accurate personalized dental care. Nevertheless, the medical community should be aware of the challenges and pitfalls that emerge to maximize the opportunities to reshape the field and improve dental care, while steering clear of the possible negative consequences.

## Declaration of competing interest

The authors declare that they have no known competing financial interests or personal relationships that could have appeared to influence the work reported in this paper.
